# Optical-Trapping Laser Techniques for Characterizing Airborne Aerosol Particles and Its Application in Chemical Aerosol Study

**DOI:** 10.3390/mi12040466

**Published:** 2021-04-20

**Authors:** Aimable Kalume, Chuji Wang, Yong-Le Pan

**Affiliations:** 1 CCDC-US Army Research Laboratory, Adelphi, MD 20783, USA; yongle.pan.civ@mail.mil; 2 Department of Physics and Astronomy, Mississippi State University, Starkville, MS 39759, USA; cw175@msstate.edu

**Keywords:** optical trapping, single particle, chemical aerosol, laser spectroscopy

## Abstract

We present a broad assessment on the studies of optically-trapped single airborne aerosol particles, particularly chemical aerosol particles, using laser technologies. To date, extensive works have been conducted on ensembles of aerosols as well as on their analogous bulk samples, and a decent general description of airborne particles has been drawn and accepted. However, substantial discrepancies between observed and expected aerosols behavior have been reported. To fill this gap, single-particle investigation has proved to be a unique intersection leading to a clear representation of microproperties and size-dependent comportment affecting the overall aerosol behavior, under various environmental conditions. In order to achieve this objective, optical-trapping technologies allow holding and manipulating a single aerosol particle, while offering significant advantages such as contactless handling, free from sample collection and preparation, prevention of contamination, versatility to any type of aerosol, and flexibility to accommodation of various analytical systems. We review spectroscopic methods that are based on the light-particle interaction, including elastic light scattering, light absorption (cavity ring-down and photoacoustic spectroscopies), inelastic light scattering and emission (Raman, laser-induced breakdown, and laser-induced fluorescence spectroscopies), and digital holography. Laser technologies offer several benefits such as high speed, high selectivity, high accuracy, and the ability to perform in real-time, in situ. This review, in particular, discusses each method, highlights the advantages and limitations, early breakthroughs, and recent progresses that have contributed to a better understanding of single particles and particle ensembles in general.

## 1. Introduction

Besides the main gaseous components (N_2_, O_2_, Ar, CO_2_, etc.), the Earth’s atmosphere includes a significant amount of fine particles (solid or liquid droplets) floating in the air, known as atmospheric aerosols. These airborne particles are typically in the size range from a few tenths of nanometers to a few tenths of microns, which can be found anywhere from the surface of the Earth up to the stratosphere, with variable composition and distribution along the vertical profile and across the global stretch, all of that on top of a pronounced temporal variability [1,2]. In general, aerosols play an important role in the atmosphere, by considerably affecting human health and urban visibility, altering Earth’s energy budget and global climate [3,4,5,6,7,8,9].

Atmospheric aerosols can be of natural origin (e.g., desert dust, volcanic ash, sea salt, clouds, pollen, fungal, spores, bacteria, etc.) [7,8,10,11,12] or anthropogenic, as a result of human activities such as fossil fuel combustion, biomass burning, mining, agriculture, cement production, etc. [5,6,9,13,14,15,16]. As opposed to bioaerosols, chemical aerosols do not have any biological material: they are rather made of condensed inorganic or organic molecules. Studies conducted over the past five decades have assessed, to varying extents, the presence of these particles in the atmosphere and have shown that they play a key role in complex heterogeneous and multiphase processes governing atmospheric chemistry. Their profound scrutiny is essential to better understand various atmospheric events, such as the scattering and absorption of incoming solar radiation or thermal radiation emitted by Earth’s surface [6,17,18,19,20], the triggering of cloud condensation or, in return, cloud droplets affecting aqueous chemistry of aerosols [4,6,21,22] and the sparking of ice nucleation [6,23,24,25,26], the generation of acid rain and its ecological consequences [27,28], the depletion of ozone, and global warming with big climate changes [29]. Chemical aerosols can heavily affect human health, cause a wide range of respiratory diseases, or act as chemical warfare agents in the worst-case scenario [30,31].

Depending on specific concerns, there are numerous methods used to measure and characterize the properties of interesting aerosol particles, such as the chemical atmospheric aerosols. Space-born methods (CALIPSO, CloudSat, MODIS, MISR, etc.) [32,33,34] utilize sensors mounted on satellites to observe global aerosol distribution; while ground-based methods (AERONET, SKYNET, MPLNET, EARLINET, etc.) [35,36,37], which comprise a collection of hundreds of sites around the world, provide complementary information to space remote sensing. Beyond large-scale distribution and abundance monitoring, a wide variety of standard and specially designed laboratory-based methods are available to study physical and chemical properties of chemical aerosols [38].

While remote sensing methods provide an overall distribution picture, a closer look is crucial in order to answer basic questions such as the detailed microproperties of a single given particle. For instance, it has been shown that atmospheric aerosols can consist of multiple phases, and each individual phase might be homogeneous or heterogeneous [39,40,41]. Those microproperties possess the ability to influence aerosol’s behavior in a particular environment by altering size and phase, evaporation rates, adsorption, absorption, surface enhanced chemistry, etc., which leads to the necessity for scrutinizing the behavior of a single particle with respect to its inherent composition [39,42,43,44].

Another benefit of studying a single particle is the ability to use non-destructive methods and perform accurate measurements on the same particle over a period of time, enabling to observe the direct response of the aerosol particle with respect to various environmental conditions (relative humidity (RH), electromagnetic radiation, presence of other chemicals, etc.). As an ultimate goal, a full and neat comprehension of a single chemical aerosol particle would help minimize systematic or random errors associated with averaged observations performed on aerosol ensembles or those arising from multistep sampling and analytical techniques.

The main intent of this review article is to highlight available capabilities, advantages and recent progress made on the characterization of chemical aerosols, within the framework of laser-based techniques performed on an optically trapped single particle. With no claim of being exhaustive, this article compiles valuable recent information scattered across the literature and portrays a general picture that serves as a point of reference to a wide scientific community, especially for scientists and researchers who are new to the field or just jumpstarting their careers.

## 2. Single-Particle Optical Trapping

In this section, we briefly describe optical trapping of a single particle within the micron size range in air. More details can be found in articles dedicated to theoretical fundamentals, in this special issue. From the pioneering work by A. Ashkin in the early 1970s [45,46], optical forces have found great use in holding and manipulating micro- and sub-micro particles in various media. The basic idea, which is at the foundation of optical tweezers, optical levitation [46,47,48], and various optical trapping schemes [49,50,51], is that a tightly focused laser beam generates strong optical force, capable of confining one or a few particles in three dimensions.

In a case where a particle size is much larger than the wavelength of the trapping laser, conditions of Mie scattering are satisfied and forces can be calculated using the simple ray optics model; when the particle is much smaller than the wavelength, it can be treated as a point dipole. However, when the particle size is comparable to the trapping laser wavelength (~0.1–10λ), which is often the case for aerosol particles, the two treatments above fail to compute optical forces accurately. The forces are rather calculated using Lorenz–Mie theory [52,53] or its improved variations (for spherical particles) and T-matrix approaches for estimating scattering properties of irregularly shaped particles.

In general, optical forces acting upon a trapped particle are divided into radiation pressure force and photophoretic force. Radiation pressure force comprises the scattering force resulting from the transfer of momentum of incident photons to the particle that push it along the light propagating direction, and the gradient force that tends to pull the particle toward the region of high intensity of the light (in the direction of the field gradient). On the other hand, photophoretic force (also known as radiometric force), which can be 4–5 orders of magnitude stronger than radiation pressure force, is due to non-uniform heat transfer on an absorbing particle. The heat transferred to surrounding gas molecules induces stronger collisions and tend to push the particle toward its cold side [49,54,55].

**Figure 1:**
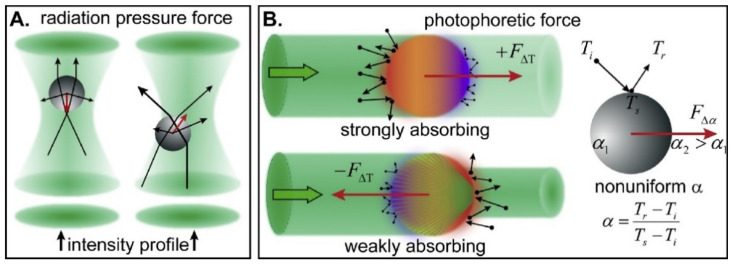
(**A**) A ray-optics description of the radiation-pressure force on a transparent sphere. (**B**) The photophoretic forces include the ΔT-force and the Δα-force. The inhomogeneous heating on strongly and weakly absorbing spheres results in positive and negative ΔT -forces. The Δα-force can exist in a particle with non-uniform thermal accommodation coefficient α, even when the particle has a uniform temperature (reprinted from [56] with permission from Elsevier, Copyright (2018) Elsevier).

All forces, described in Figure 1, can act simultaneously on a particle. Their respective influences depend on the nature of the particle, the trapping beam configuration, and the trapping medium. For a homogeneous spherical particle, the three forces can be estimated as follow [56,57]:

$$F_{scattering}=\frac{I_{o}}{c}\;\frac{128\pi^{5}r^{6}}{3\lambda^{4}}\left(\frac{n_{rel}^{2}-1}{n_{rel}^{2}+2}\right)n_{med}$$

$$F_{gradient}=-\frac{n_{med}^{3}r^{3}}{2}\;\left(\frac{n_{rel}^{2}-1}{n_{rel}^{2}+2}\right)\alpha \nabla E^{2}$$

$$F_{photophoretic}=-J_{1}\frac{9\pi \mu_{a}^{2}rI_{o}}{2\rho_{a}T\left(\kappa_{p}+\kappa_{a}\right)}$$

where *I_o_* is the incident beam intensity, *λ* is the laser wavelength, *r* is the particle’s radius, *n_part_* and *n_med_* are the refractive index of the particle and the medium, respectively, *n_rel_* is the relative refractive index, and *n_rel_* = (*n_part_/n_med_*), α is the real part of the polarizability, E is the optical field, *J*_1_ defines the axial direction of the photophoretic force, *T* is the temperature, $\kappa_{p}$ is the thermal conductivity of the particle, and *µ_a_*, *κ_a_*, and *ρ_a_* are the viscosity, thermal conductivity, and mass density under normal conditions of temperature and pressure.

In order to trap a particle in a three-dimension space, the resulting optical forces pulling the particle toward the laser beam focus have to exceed all other forces driving it away, such as scattering force, excessive photophoretic force, drag force (fluid resistance), gravitational force, and convection force, or forces from any other random disturbance on the particle.

**Figure 2:**
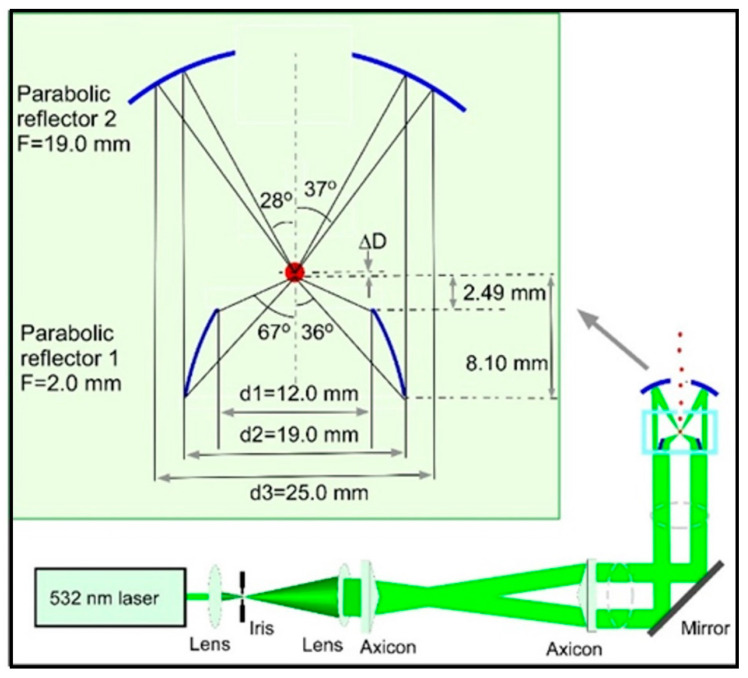
Schematic of the optical trapping apparatus with the detailed alignments and dimensions of the two focusing parabolic reflectors. (Reprinted with permission from [50] under Open Access License. Copyright (2019) The Optical Society of America).

Depending on the type of particle, the light source, the surrounding medium, and the associated instrumentation, there are numerous ways to arrange optical trapping systems, which can be grouped into traps for absorbing particles, for non-absorbing, and for universal traps. Representative configurations, for a non-absorbing particle, include a single-beam radiation-pressure trap in which an upward-directed laser beam provides simple levitation of a particle by balancing the gravitational force [46]. This design is improved in optical tweezers by using a high NA objective to achieve a large gradient force at low laser power, resulting in a 3D confinement [57,58,59]. In principle, most of trapping schemes are compatible with trapping particles in air. However, a higher relative refractive index in air than in liquid results in larger scattering forces, which tend to destabilize the trap. One way to resolve this problem is to utilize high numerical aperture objectives, but these have the inconvenience of limited working distance and are challenging for the accommodation of other observation components. A better option is to use two counter-propagating beams (vertical or horizontal) focused by two objectives of relatively lower NA, with the chance of balancing individual scattering forces [60,61]. For an absorbing particle, a single-beam can easily achieve photophoretic trapping, regardless of the propagation orientation [54,62,63]. Due to thermal effects at the interface, the trapped particle tends to undergo Brownian motions that may affect measurements and observations [64]. The stability can be improved by choosing a beam profile that presents regions of low intensity, such as a focused hollow beam [55,65] or other exotic beams such as optical vortex, doughnut beam, Laguerre–Gaussian beam, bottle beam, speckle field, annular beam, etc. [66,67,68,69,70,71,72,73,74,75]. A dual-beam photophoretic trap is attained by addition of a second counter-propagating beam, which, for instance, increases trapping robustness by holding an absorbing particle at the low-intensity center of hollow beams or vortex beams [76,77]. Note that dual-beam configuration does not require an additional laser source or necessarily translate into duplication of every optical component used in the setup and alignment issues associated with, since the dual beams can be generated in the late stages by splitting the main laser beam, using a beam splitter. Further improvements on the stability, simplicity, and instrumentation cost are obtained by using a confocal-beam photophoretic trap, where a sole focused trapping beam is reflected back by a concave mirror to form a virtually counter-propagating optical field [51,56]. As the previously described two categories were particle’s type exclusive, there was a need for an optical trap suitable for a wider range of particles, regardless of their composition, absorptivity, size, and morphology. Efforts in this direction led Carruthers and co-workers to design a counter-propagating Bessel beams trap that worked for both liquid droplets and weakly absorbing pollens [78]. In 2015, Redding and co-workers [49] revealed a first and more comprehensive universal optical trap (UOT), which worked with absorbing or non-absorbing particles, irrespective to their morphology. This configuration uses a single hollow beam having a low-intensity center region for photophoretic trapping of absorbing particles while simultaneously reducing the scattering force near the focal point, to trap non-absorbing particles by means of radiation pressure force, using a relatively low NA optic (~0.55). Beyond the focal spot, the expanding hollow beam reflects back from a highly reflective mirror to generate a virtual second beam, offering more stability and stiffness to the trap. The latest state-of-the-art universal trapping system was designed and built in 2019 by Pan and co-workers, as seen in Figure 2 [50] using a single hollow beam reflected by two parabolic reflectors to generate two counter-propagating conical beams (two focal points), independently adjustable along the axis of propagation. This system demonstrated high efficiency in trapping various particles (arbitrary in nature) in air, with size ranging from 30 µm down to 1 µm and below. This system offers several appealing advantages including high efficiency (>50% for non-absorbing and ~100% for absorbing particles), improved robustness, simplicity in design, minimum optics contamination by keeping the particle away from optical surfaces, and a large free space for easy accommodation of various analytical techniques and spectroscopic measurements.

## 3. Single-Particle Laser-Based Characterization

Aerosol particles have a highly complex nature and behavior, and they play a role in a wide range of disciplines involving a variety of expertise. Therefore, it is unrealistic to obtain a complete description using a single technique or instrument. This section highlights a few characterization methods that are, fundamentally, based on the “particle-light interaction”.

When an aerosol particle interacts with an incident light beam, photons can be reflected, refracted, absorbed by the particle and re-emitted at different wavelengths, or simply scattered at the same wavelength but in all directions. Being dependent on the particle’s nature and following different sets of fundamental selection rules, these processes offer reliable laser-based methods to investigate particles with significant benefits such as high speed, enhanced selectivity, accuracy, high reproducibility, ability to perform in real-time in situ, and avoiding problems associated with other sampling and analytical techniques (instrumentation, filtration, storage, transport, sample preparation, chemical reaction, etc.).

However, similar to the other experimental methods, the optical-trapping laser techniques discussed in this article have their own particular challenges and limitations. We will discuss them in each section below.

### 3.1. Elastic Light Scattering (ELS) Characterization

Elastic light scattering is a non-invasive diagnostic technique based on the phenomenon of incident photons scattering from a material, without change in frequency (photon energy). For particles with a diameter much smaller than the incident wavelength (Rayleigh scattering regime), the light is scattered nearly symmetrically in the forward and backward directions and the scattered intensity is proportional to the sixth power of the particle diameter and inversely proportional to the fourth power of the wavelength ($I\propto d^{6}/\lambda^{4}$). For particles with size larger or commensurable with the wavelength (Debye or Mie scattering regime), the local electric field is no longer uniform: there is a strong particle–light interaction resulting in an asymmetrical scattering intensity (angle-dependent), with no simple direct relation to the diameter [38,79,80].

In practice, elastic light scattering is a flexible technique for a single-particle characterization, due to the versatility in selection of illumination sources and associated detection devices, which can be, independently, arranged outside the trapping chamber. Typically, the measurement can be obtained from a single or several discrete signals [81,82] recorded with photodiodes and photomultiplier tubes (PMTs) or as a 2D scattering pattern over a large solid angle, often recorded by a charge-coupled device (CCD). For angular-resolved measurement, the detector can be mounted on a rotating mount or substituted by an array of detectors around the trap [82,83,84].

In most of optical trapping systems, a sudden increase in measurable elastic light scattering indicates the presence of a particle in contrast to the low-intensity background in the absence of a scatterer. Measured with highly sensitive detectors, elastic scattering can provide information on the position and motion, which in return is used for trapping stability via feedback processes [85,86,87,88]. However, the focus of this section is the role of elastic light scattering in quantitative characterization of chemical aerosols.

Elastic light scattering from small particles has an extremely large scattering cross-section, enough to overcome the sample scarcity, with a relatively high signal-to-noise ratio, making it measurable at a trace level or for a single-particle. In the Mie regime, where the particle size is commensurable with the light wavelength, the intensity of elastic scattered light, for a given solid angle, is a direct function of the incident intensity, particle size, shape, surface roughness, refractive index, and chemical composition. Experimentally, with some of these properties known, elastic scattering can be used to determine the remaining properties with high accuracy.

In 1977, Ashkin and Dziedzic [89] measured the variation of radiation-pressure force on transparent dielectric spheres with wavelength and size. They observed sharp resonance peaks appearing on top of the scattered intensity profile, due to the total internal reflection when the dielectric sphere (or droplet) acts like a low-loss optical cavity. These resonances were used to precisely determine the size of particles, improving the accuracy with at least two orders of magnitude in comparison with far-field scattering techniques available at that time. The findings in this experiment laid the groundwork for similar experiments in which they demonstrated the use of resonances for high-precision measurement of sphere size, refractive index, temperature, and vapor pressure [90]. Optical levitation was also used by Grehan and Gouesbet to study the theory of the quasi-elastic scattering from an individual droplet instead of clouds [91]. A good agreement was found between experimental results and theoretical simulations for a droplet of 29.5 µm in diameter and 1.5-0*i* refractive index, and it was confirmed with microscopic measurement of 29 ± 1 µm. However, in order to strictly apply Lorenz–Mie theory, they suggested use of an independent illumination beam rather than the one used for trapping in future studies.

**Figure 3:**
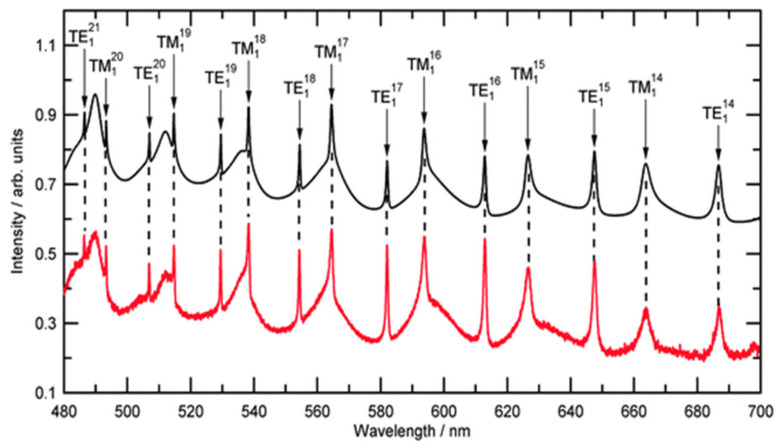
A typical recorded experimental Mie spectrum for an optically trapped polystyrene bead is shown in red. The calculated Mie spectra for a polystyrene bead of size 1.227 μm, and a dispersion of refractive index, are shown in black and are offset by 0.5 arbitrary units (reprinted from [95] with permission under Creative Commons Attribution license, Copyright (2013) The Royal Society of Chemistry).

Ever since, the use of Mie resonance peaks to retrieve physical properties of optically trapped particles has grown in popularity and been readily adapted to the development of new trapping configurations [70,92,93,94]. Using a longitudinal optical trapping scheme, Carruthers et al. [70] determined the size of aerosol particles from the angular variation of the elastic light scattering. Measurements were performed independently and simultaneously from two orthogonally polarized trapping beams and a third Gaussian probe beam. Flexibility in this setup also allowed to observe and characterize coalescence of two droplets (one bound, another guided) in accordance to Mie theory calculation. Jones and co-workers [95] analyzed backscattered light from forty-six polystyrene beads and deducted radius and new empirical constants for the wavelength dispersion of refractive index in air (Figure 3). Finding that each polystyrene bead had a different radius and refractive index led them to caution the use of a monodisperse value of refractive index for commercial samples while calibrating instruments for high accuracy.

In general, accurate interpretation of ELS spectra has always heavily relied on data fitting and theoretical simulations. Extensive efforts in predicting the behavior of particles in the presence of light have resulted in a large number of computational models. The details can be found elsewhere in the literature, including those based on Rayleigh scattering and Mie theory and their variants [80,96,97] as well as methods built upon the T-matrix approach, compiled in a database of theoretical works, periodically updated [98,99,100,101,102,103,104,105]. Several algorithms used to analyze ELS phase function or patterns [106,107,108,109,110] perform by varying the radius of the particle and the refractive index values, in order to minimize the difference between the observed and calculated data until the best fit is achieved, although this process tends to be computationally expensive. Preston and Reid have shown that once the mode assignment is known, the parameters of the best fit can be, instantaneously, found by solving a system of linear equations [111].

While the idea discussed above appears straightforward for pure substances, interpretation of ELS in multicomponent and multiphase particles is often challenging due to the complications associated with the distribution of the resulting refractive index and how it may change during evaporation, in case of trapped droplets. Although analyzing Mie resonance peaks in multicomponent particles can be computationally expensive, this approach can serve to study evaporation, growth, and diffusion in complex particles, and several researchers have investigated morphology-dependent resonances (MDR) in layered droplets [112,113,114,115,116,117].

Unlike dielectric spheres (e.g., aerosol droplets), for which the wavelength- or angular-resolved light scattering works unambiguously, non-transparent and arbitrarily shaped solid particles often require a better description of multidimensional patterns using the scattered light at various polar and azimuthal angles, which is often referred to as two-dimensional angular optical scattering (TAOS). Numerous theoretical models [118,119,120,121,122] have calculated the scattering from highly irregular and heterogeneous particles. However, in the scarcity of experimental studies, it exposes the challenge of measuring properties that are orientation dependent, with instrumentation difficulty such as in measuring a back-scattering polar angle at θ = ~180°. Pan and co-workers [123] managed to record the scattering patterns from a single laser trapped aerosol particle, over the backward hemisphere, with high reproducibility over minutes to hours.

In 2016, Fu and co-workers [124] combined direct imaging and a back-scattering measurement covering a polar angle θ = 167.7–180° (including the exact 180°) and an azimuthal angle ø = 0–360°. They demonstrated how scattering patterns change with the particle size, shape, surface roughness, or orientation. This dependency, especially for the orientation, since even the same particle can have an unlimited number of patterns, has been the limiting factor in using elastic scattered patterns as a major characterization method for unknown aerosol particles. Fortunately, recent seminal works by Piedra et al. [125,126] applied machine learning (ML) algorithms to sets of computationally generated scattering patterns and achieved particle classification into groups, with an accuracy of ~70% for regularly shaped and ~90% for irregularly shaped particles. Ongoing efforts devoted toward advanced instrumentation for scattered light, improved and stable trapping techniques, as well as reliable ML algorithms, give hope to use elastic light scattering as a practical tool for fast characterization of aerosol particles.

Although ELS has a high optical cross-section to obtain strong scattering signals, used to supply morphology information (size, shape, etc., of physical properties) and refractive index (containing some chemical property) of the trapped single particles, it provides limited specific information about the chemical composition of the particles. Therefore, mostly ELS is used as a supplement tool in single-particle study.

### 3.2. Light Absorption Spectroscopy

#### 3.2.1. Cavity Ringdown Spectroscopy (CRDS)

Cavity ringdown spectroscopy (CRDS) is a laser absorption spectral technique used to measure optical extinction of the light (loss due to absorption and scattering) in the path between two high-reflectivity mirrors. In this setup, the light bouncing back and forth between the mirrors mimics a long effective pathlength (up to tens of kilometers) leading to an extremely high sensitivity, suitable for trace gas measurements [127,128]. This spectroscopic technique has been applied to measure extinction coefficients (σ_ext_) of aerosol ensembles and gases, which is later used to retrieve refractive index [129,130,131,132,133,134,135]. However, the reliability of this approach has some limitations, since it implies some degree of assumption on the particle size distribution within the ringdown beam.

Conversely, in single-particle cavity ringdown spectroscopy (SP-CRDS), where the size is trivially determined from elastic scattering, a particle trapped inside the cavity is allowed to interact with a probe beam at each of its multiple trips between highly reflective surfaces, and the attenuation time or ringdown time (RDT) is measured. This method proved to be highly accurate in comparison to other ensemble measurements. Typically, the extinction coefficient is given by:

$$\sigma_{ext}=\frac{L\pi \omega^{2}}{2c}\;\left(\frac{1}{\tau }-\frac{1}{\tau_{0}}\right)$$

where *L* is the distance between the reflecting surfaces, *ω* is the beam’s waist, *c* is the speed of light, *τ* is the ringdown time with sample in, and *τ*_0_ is the ringdown time of an empty cavity [136].

**Figure 4:**
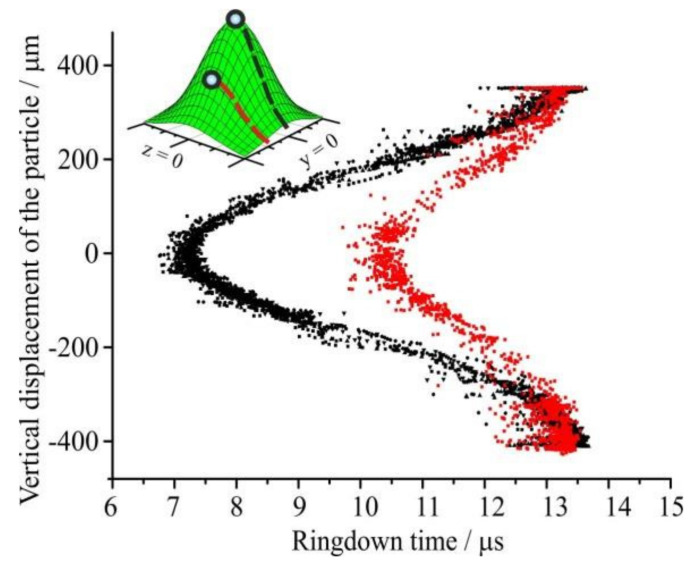
Sensitivity of the measured RDT to the position of a 1 µm radius particle within the CRDS beam. The change in RDT time with vertical position is recorded for a particle passing through the center of the beam (black symbols) and ~200 µm off-center (red symbols) (reprinted with permission from [137]. Copyright (2013) American Chemical Society).

Walker and co-workers [137] reported measurements of light extinction from a single aerosol particle, trapped in a Bessel beam, where they explored dynamics of evaporation and quantified the change in optical cross-section for an aqueous sodium chloride droplet. Since the sensitivity of the extinction time is directly related to the CRDS laser intensity (Figure 4), it is critical to place and maintain the particle at a known location of the highest intensity—a challenge resolved by using a feedback system for size-changing particles such as evaporating droplets. This can be achieved by constantly adjusting the trapping point in space to match the highest intensity across the CRDS beam.

**Figure 5:**
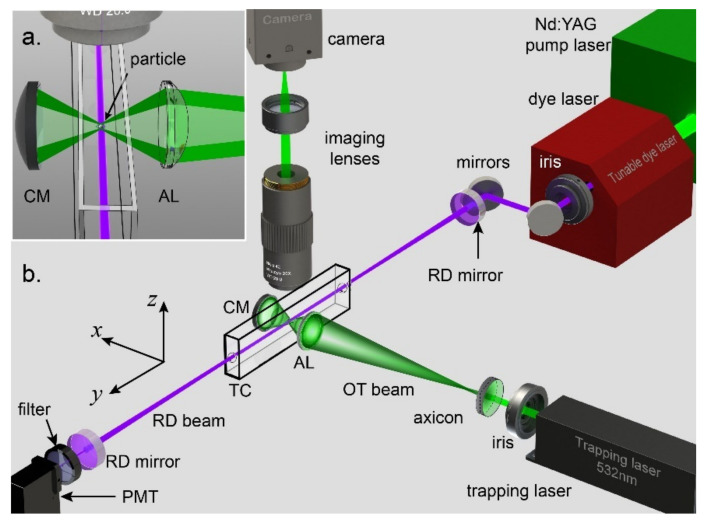
Experimental scheme of the optical trap-cavity ring-down spectroscopy (OTCRDS) system. CM: concave mirror, AL: aspheric lens, RD mirror: ring-down mirror, RD beam: ring-down beam, OT beam: optical trapping beam, TC: trapping cell, PMT: photomultiplier tube. The inset figure illustrates the detailed optical field near the trapping zone. (Reprinted with permission from [139] under Open Access Publishing Agreement. Copyright (2017) The Optical Society of America).

In 2015, Wang et al. [138] demonstrated an optical trap-cavity ringdown (OT-CRDS) scope, enabling to view the changes of physical and chemical properties via light extinction measurements, in addition to routine size, motion, and restoring forces, that was estimated as small as ~10^−10^ N/m. Two years later, the same group [139] improved their system with a more robust trapping scheme, based on confocal hollow beams, as shown in Figure 5. Scanning a monowall carbon nanotube across the CRDS beam yielded an extinction efficiency of *Q_ext_* = 1.96 and the system was sensitive enough to distinguish the number of particles in the trap. By studying several kinds of aerosol particles (with similarities and differences) under various conditions, they showed the need for considering the compound effect of morphology and composition on the scattering and absorption components in the total extinction, in addition to the size-, material-, and wavelength dependence of optical extinction.

In general, the particle extinction cross-section σ*_ext_*, and therefore the interaction of light with an aerosol particle, depends on the scattering cross-section σ*_sca_* and the absorption cross-section σ*_abs_*, which are, respectively, functions of *n* and *k* components of the refractive index (*m = n + ki*). Cotterell and co-workers [140] developed simulation methods to assess refractive index retrieval accuracies from the extinction cross-section of a single particle trapped in a Bessel beam. The level of accuracy obtained for particles of diameter >1 µm is sufficient to calculate reliable aerosol radiative forcing efficiency, a key quantity used in modeling Earth’s climate.

In an elegant work by Cotterell et al. [136], the comprehensive σ*_ext_* and phase-function measurements were performed on a series of aqueous droplets of inorganic salts commonly found in the atmosphere (NaCl, NaNO_3_, (NH_4_)SO_4_, NH_4_HSO_4_, and Na_2_SO_4_), and a complete parameterization of the relative humidity (RH) and wavelength dependence of the refractive index (RI) of the studied droplets was reported. Overall, the results showed better accuracy in RI retrieved from σ*_ext_* in comparison with phase function (PF) retrieval, with standard deviation values in a ratio of 1:2, respectively. They also highlighted the improved precision in using a Gaussian-profile illumination beam in PF measurements, instead of a focused Bessel beam.

A recent work by Alali et al. [141] utilized optical-trapping CRDS to study simulants of extraterrestrial dusts (Martian and Lunar) and other inorganic particles in the ultraviolet wavelength (308 nm). Despite pronounced differences in sampled particles (density, shape, chemical composition, tendency to aggregate, size range (4–50 µm), absorptivity, etc.), a flexible universal optical trap was able to handle all particles efficiently. Stability of this system was established by a high level of reproducibility in measurements taken when the particle was optically moved in and outside of the CRDS beam. Note that since measured extinctions strongly depend on size and morphology, a reliable material dependence analysis had to take into account geometrical cross-section and calculated extinction efficiencies as Q_ext_ = σ_ext_/σ_geom_ [141].

While CRDS has a very high sensitivity for obtaining absolute optical extinction, usually it is used in studying gaseous materials which absorb light at specific wavelengths, and in turn to determine mole fractions down to the parts per trillion level. For the trapped single particle, the absorption from CRDS can be extracted from the total extinction by precisely measuring the scattering intensity in comparison with the theoretical simulation, particularly for these well-defined particles, e.g., microspheres, but it is difficult to decouple the absorption and scattering information for the irregularly shaped particles. In general, CRDS requires a precise alignment in order to achieve the mode-matching condition, and the instability of any component, in the experimental setup, would reduce the signal quality significantly.

#### 3.2.2. Photoacoustic Spectroscopy

Photoacoustic (PA) spectroscopy is an opto-thermal technique used to measure periodic temperature fluctuations in the sample as pressure fluctuations. In the PA process, an incident photon energy absorbed by molecules is converted, via radiation-less relaxation, into thermal energy. The resultant heat is dissipated to the surrounding gas and induces pressure waves that can be measured by an acoustic detector (generally a microphone mounted inside the resonance chamber or a tuning fork for better sensitivity).

Before applications to a suspended particle, an early study by Yoshinaga et al. [142] showed the quantitative analysis potential of laser-induced photoacoustic spectroscopy applied on a single aerosol particle resting on a quartz plate. However, the PA signal suffered unsolicited contributions from other materials existing in the adsorbent in the experiment.

**Figure 6:**
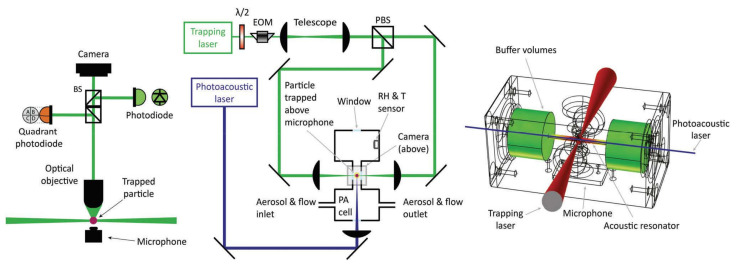
Experimental setup used for single-particle photoacoustic spectroscopy. **Left:** Side view of the optical setup collecting scattered light from the particle and projecting it onto a quadrant photodiode, a camera, and a photodiode. **Center:** Top view of the optical table comprised of a trapping laser (green), photoacoustic laser (blue), the PA cell, and a trapped particle (red). The trapping laser is expanded using a telescope and the relative intensities of the two trapping beams are controlled by the electro-optic modulator (EOM). **Right:** The photoacoustic cell used in these experiments showing the trapping laser (red), the photoacoustic laser (blue), buffer volumes, and a microphone. The color scheme inside the cell represents the acoustic intensity originating from the particle. (Reprinted with permission from [144], under Creative Commons Attribution License. Copyright (2019) The Royal Society of Chemistry).

New designs allowing standard particle trapping inside a PA cell have been developed and an example of a scheme combining optical trapping and PA spectrometer is shown in Figure 6. Note that such setups require a stable trapping, strong enough to overcome photophoretic forces arising from unevenly heating the particle. Cremer and co-workers [143] performed photoacoustic measurements on single laser-trapped droplets and achieved a minimum detectable absorption coefficient of α_min_ = 0.0074 × 10^−6^ m^−1^.

Since a large absorption would, eventually, lead to evaporation and/or photolysis, a careful analysis must account for photokinetics associated with material loss and size reduction (measured by elastic scattering) as well as for intensity nanofocusing throughout the droplet. Theoretical models have predicted size-dependent damping of the PA signal due to the rise in thermal inertia as the particle size increases [145,146,147,148]. This prediction was, unambiguously, confirmed by Cremer et al. [149] by measuring a time-resolved PA signal of an evaporating droplet of dye-doped tetraethylene glycol (Vis441/TEG) over a period of 1 h. During this monitoring time, both PA signal and overall elastic scattered light intensity diminished as a result of ~95% particle volume loss. On the other hand, regarding the RH correlation to the PA signal, previous different studies on aerosol ensembles have reached contradictory conclusions, whether the PA signal increases [150] or decreases with RH [148,151,152]. This controversy was settled by Diveky et al. by assessing the PA signal of an optically trapped single droplet of tetraethylene glycol (TEG) under varying RH [144]. The PA signal consists of two measurable components: the amplitude (PAA) and the phase (PAP). It was found that change in the contribution of the heat flux and mass flux induces a PAS-RH correlation, where the PA signal is proportional to the RH for particles larger than ~2.6 µm, while the trend is reversed for small particles (<2.1 µm). A more recent fundamental investigation by the same group [153] analyzed PAA and PAP using elastic scattering by means of modulated Mie scattering (MMS) which allowed quantification of individual contributions of heat flux, mass flux, and volume change. The measurement was proven to be more sensitive in PAP than in PAA with respect to mass flux, and it led to a suggestion that PAP measurement would be more appropriate in the study of water evaporation or uptake in aerosol particles. These findings, particularly, highlight the need for studying a single particle when it comes to understanding the fundamental processes governing aerosol ensembles.

Since the absorption is derived from measuring the sound wave generated by the change of thermal pressure under the illumination of periodically modulated or pulsed light source, it would be practical if the trapping light source uses another wavelength out of the absorption region. The pulsed illuminating light at the absorption wavelength could also generate very strong PPF, which is capable of disturbing the particle or pushing it away from the trapping position. Therefore, the trapping must be strong enough to hold the particle at a fixed position.

### 3.3. Inelastic Light Scattering—Light Emission Spectroscopy

#### 3.3.1. Raman Spectroscopy

Raman spectroscopy is an in situ non-invasive laser technique based upon the interaction of light with the material, where a small portion of the incident light is scattered with different photon energy (or wavelength). Raman spectroscopy is mainly used to investigate detailed molecular composition of various chemicals, since the frequency shifts observed in the spectrum correspond to the molecular ro-vibrational level energies, inherent to a molecular configuration.

Single-particle trapping allows a long acquisition time to overcome the difficulty in measuring the relatively weak Raman signal, since only ~0.1% of the scattered radiation is Raman scattering [154], the rest being scattered without change in frequency and therefore providing little information about the molecular composition. A typical experimental setup finds the simplicity of coupling single-particle trapping to Raman spectroscopy, which only requires an excitation laser source, optics to collect the scattered light and a detection system.

**Figure 7:**
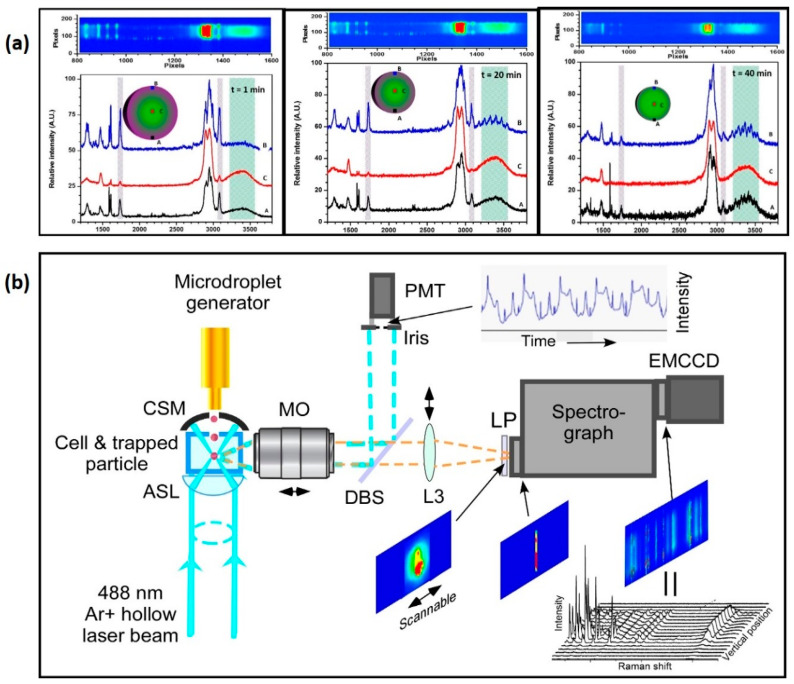
(**a**) Spatially resolved Raman spectra revealed a typical core-shell morphology, with glycerol-rich core, coated with a layer of DEP. Time-resolved spectra showed that DEP evaporated faster than the less volatile glycerol, however, both chemicals in the mixture evaporated at faster rates than within their own pure droplets. (**b**) Experimental setup for measuring position-resolved temporal Raman spectra and particle size from a laser-trapped single airborne aerosol particle. ASL: aspheric lens; CSM: concave spherical mirror; DBS: dichroic beam splitter; L: lens; LP: long-pass filter; M: mirror; MO: microscopic objective; PMT: photomultiplier tube. (Reprinted and adapted from [39], with permission under Creative Commons Attribution License, Copyright (2018) The Royal Society of Chemistry).

As highlighted in Figure 7, continuous technological advances have allowed substantial improvements, in terms of more robust trapping schemes featuring position feedback, better signal collection with high-quality filters to cut off the excitation wavelength, and highly sensitive detection systems (CCD, image-intensified CCD (ICCD), electron multiplying CCD (EMCCD), photomultiplier tube (PMT), or avalanche photodiode (APD)).

Early in 1976, before the development of stable trapping devices, Cheung and co-workers [155] had recorded Raman spectra of CH_3_I single particles in optically isotropic solvent (isopentane and CCl_4_). However, it was not until 1984 that Raman spectra were measured from optically levitated glass spheres, by Thurn et al. [61] and from microdroplets in optical trap, by Knoll et al. [156].

As a non-destructive technique, time-resolved Raman spectroscopy has proven to be very valuable for monitoring physical–chemical property changes (evaporation [41,157,158], hygroscopic growth, deliquescence [41,158,159], efflorescence [41,158,159], chemical reactions [158,160,161,162]) occurring in trapped single aerosol particles. Note that, while phase separation studies have flourished in the case of organic–inorganic mixed droplets [43,158,163,164,165,166], there are few reports on organic–organic droplets, where Raman spectroscopy is impeded by similarities in vibrational modes for various organic compounds, resulting in low chemical specificity in terms of Raman shift.

As shown in Figure 7, our group [39] combined position- and time-resolved Raman spectroscopy with optical trapping to demonstrate direct observation of phase separation within multicomponent droplets of diethylphtalate (DEP) and glycerol. Vertically resolved Raman sampling was performed through a narrow slit and scanning it across the droplet resulted in a complete position-resolved Raman scattering measurement, revealing a neat core-shell morphology with glycerol at the center, coated with a layer of DEP. Continuous measurement over time allowed to monitor the evaporation dynamics of an organic–organic mixed droplet, without the challenge of spectral overlap normally found in an averaged single measurement.

A recent study by David et al. [157] followed the real-time composition change in an e-cigarette aerosol droplet trapped in optical tweezers, under controlled RH. Time-resolved Raman scattering was used to monitor the respective contributions of four main chemical components within the e-cigarette (nicotine, propylene glycol, vegetable glycerol, and water). The initial mass loss is dominated by fast evaporation of propylene glycol, dropping from 68% to 20% within 20 s. This stabilization at low concentration corresponds to significant intermolecular interactions between the remaining propylene glycol and other components (e.g., vegetable glycerol). It was also found that nicotine behavior depends on the pH of the droplet, where it evaporates completely under basic conditions (pKa = 7.9); while under acidic conditions (pKa = 3.2), its nonvolatile protonated form lingers longer in the droplet.

In the early studies [86,167,168,169,170], sharp resonance peaks resulting from light amplification within a high-Q cavity, were observed in a Raman spectrum of optically levitated microdroplets, superimposed to spontaneous Raman peaks. The existence and position of these MDRs, also known as whispering gallery modes (WGMs), are predicted by Lorenz–Mie theory [108] with high precision, and they can serve to accurately determine the particle physical properties [110,171,172,173,174]. At wavelengths commensurate with WGMs, spontaneous Raman photons can be subject to total internal reflection within the droplet, resulting in stimulated Raman scattering (SRS). Since the spontaneous Raman signal is weak, the signal intensity can be dramatically enhanced by non-linear phase-matching processes such as SRS, coherent Stokes Raman spectroscopy (CSRS), and coherent anti-Stokes Raman spectroscopy (CARS). Following the first observation of SRS from an individual water droplet by Snow et al. in 1985, it has been shown that by deliberately pumping the sample in such way that the energy difference between incident photons and spontaneous Raman photons corresponds to a frequency of Raman active mode, its intensity can be enhanced by orders of magnitude [175,176,177]. These MDR peaks provide a good indicator on the phase distribution, since they are quenched by uneven distribution in refractive index (inhomogeneous and partially engulfed mixed droplets) [178], yet they still exist in a phase separation resulting in core-shell morphology, and Gorkowski et al. [172] proposed an algorithm to fit unlabeled WGMs in a multicomponent droplet. During volume change in a liquid particle, WGMs blue-shift with evaporation and red-shift with the growth of the droplet. However, this may impede accurate quantitative retrieval of the concentration using the spontaneous Raman peaks.

Due to the fact that the RS signal from a single trapped particle is relatively weak, the detection devices and the alignment of the whole system have much higher requirements, and that could be a big challenge in the experiment. One elegant part of the OT-RS is that the same laser beam can be used for the trapping and for Raman excitation. If two lasers at different wavelengths were used, it would be wise to use a trapping laser in the near-infrared, which should be far away from the Raman spectra region generated by the Raman excitation laser. As discussed above, another challenge is the peak overlapping in Raman spectra of multicomponent particles, but this can be solved by performing position-resolved measurements on the particle.

#### 3.3.2. Laser-Induced Fluorescence (LIF) Spectroscopy

By definition, fluorescence is a luminescence phenomenon due to radiative relaxation back to the ground state of a molecule that was previously excited to a higher electronic state by absorption of incoming photon energy.

Despite low specificity rising from broad and sometimes ambiguous spectral bands, fluorescence spectroscopy has been very common to study biological specimens and bioaerosols, thanks to the abundance of fluorophores—molecular fragments responsible for fluorescence emission. However, its application to chemical aerosols has been limited to few studies by the scarcity of organic material, which translates into a weak signal further undermined by single chemical aerosol characterization.

Kuriakose and co-workers [179] managed to trap nonfluorescent and fluorescent microspheres in an evanescent field using a femtosecond laser (780 nm and 80 MHz). They found that the fluorescence spectrum in the near-field setup was four times stronger than in the far-field. In fact, the fluorescence intensity showed a quadratic dependence on the excitation power, resulting from a two-photon excitation under fast illumination. Bhatt et al. [180] used optical forces to trap various polystyrene beads. They had trouble with trapping fluorescent particles, with quasi impossibility when the maximum excitation wavelength was too close to the trapping laser, 540 nm and 532 nm, respectively. Upon longer exposure to the laser light, trapping stability started to improve gradually, accompanied with decrease in the fluorescence signal. This issue, one of the limiting factors for applying fluorescence spectroscopy on optically trapped absorbing particles, could be explained by the fact that the typical trapping system used was suitable for non-absorbing particles.

**Figure 8:**
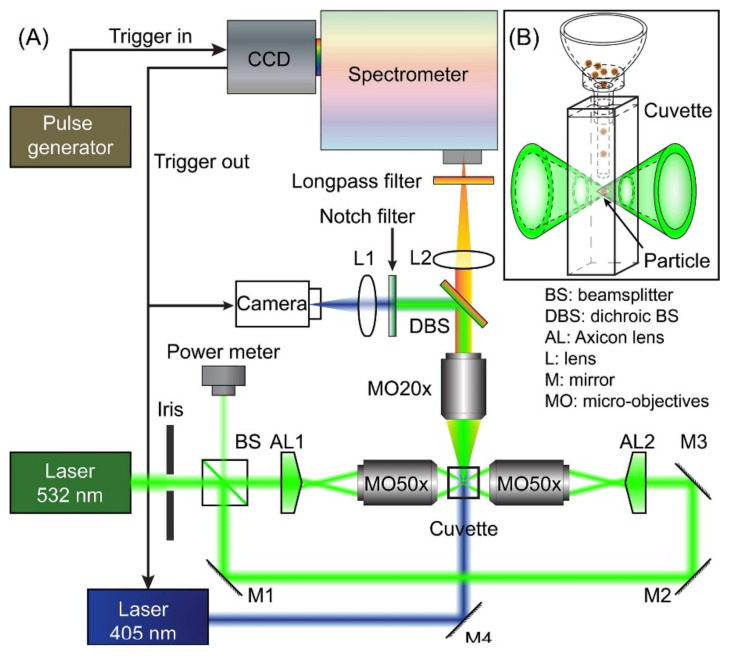
The experimental setup. (**A**) The OT-RS system based on two counter-propagating hollow beams for trapping and characterizing both absorbing and transparent particles. (**B**) The detailed particle introduction setup and the hollow optical trap. (Reprinted with permission from [181]. Copyright (2107) Elsevier).

**Figure 9:**
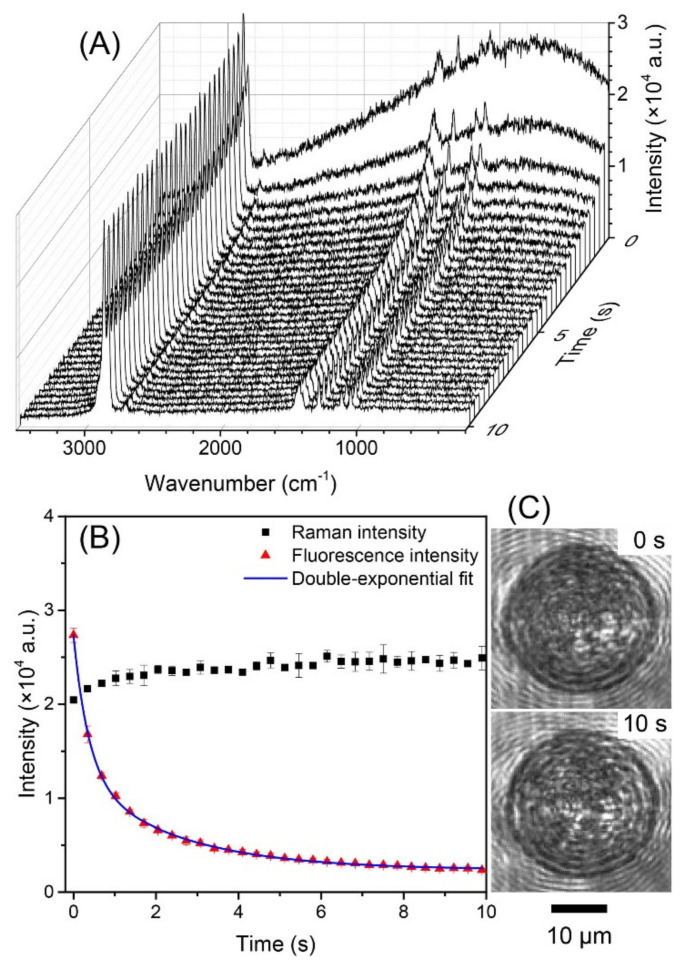
The evolution process from fluorescence to Raman spectra of a single strongly absorbing rhodamine-B-doped PE microsphere in the optical trap. (**A**) The time-resolved spectra in the first 10 s. (**B**) The fluorescence intensity (red triangles) was fitted to a double-exponential decay (blue solid line). The intensity of the Raman peak at ~2890 cm^−1^ (black squares) remained relatively constant. (**C**) Images of the trapped particle taken at 0 s and 10 s. (Reprinted with permission from [181]. Copyright (2107) Elsevier).

As previously discussed in Section 2, the universal trapping system [49] can trap both absorbing or non-absorbing particles. A similar system (Figure 8) was used by Wang’s group [181] to trap polyethylene beads. When beads coated with fluorescent green or Rhodamine B dyes were trapped, the initial Raman spectrum was overwhelmed by a strong laser-induced fluorescence. Unlike in the previously discussed study, this system withstood photophoretic disturbances, kept the particle stable in the trap, allowed a fast photobleaching of the fluorophores, and left behind a clean Raman spectrum, as shown in Figure 9. Note that, due to an effective heat dissipation in air, the photobleach can be achieved within seconds and without damage to the particle, unlike as it is for particles resting on substrate.

Spectrally narrow (0.1 nm) resonance peaks analogous to the MDR previously discussed in the light scattering section were also observed in fluorescence spectra, such as ethanol/fluorescent dye, and they were used to calculate evaporation rates of ethanol in a stream of droplets, in an early work by Tzeng et al. [182]. Later in 2001, Pastel and Struthers [183] performed similar experiments on ethylene glycol/Rhodamine-590 droplets, this time trapped in counter-propagating beams. An 800 nm diode laser was used to avoid sudden photobleach, while a weaker 532 nm laser was used for fluorescence excitation in droplets, and the emission spectra were recorded around 560 nm. Fitting of first-order MDRs yielded evaporation rates and reliable particle size (e.g., accuracy of 1% near size parameter 27).

Thompson and co-workers [184] studied fluorescence spectra of dye-doped bisphere (made of beads of equal diameters) and they demonstrated coherent coupling of axial modes, by making separation-sensitive measurements where observed MDRs’ mode splitting decreased when the spacing between the individual beads increased. However, no splitting was observed for particles of disproportionate diameters with no wavelength overlap between widths of MDRs in their spectra, resulting in negligible coupling regardless of the spacing.

In general, fluorescence has a relatively strong signal from micron-sized particles; however, its application to chemical aerosol characterization has limitations due to the lack of fluorophores in most chemical aerosols. A strong fluorescence excitation may lead to photolysis and particle damage, affecting other subsequent observations for the same particle, unless when photobleaching is needed to obtain a good signal-to-noise ratio Raman spectra in case of OT-RS characterization.

#### 3.3.3. Laser-Induced Breakdown Spectroscopy

Different from the molecular spectroscopic procedures discussed in the previous sections, laser induced breakdown spectroscopy (LIBS) is a type of atomic emission technique using laser power to instantly break the matter into its elementary components. Analysis of the recorded atomic emission spectrum provides fast chemical characterization, suitable for atmospheric aerosols, where elemental composition can bring to light rich information on aerosol makeup, source, and toxicity. With the purpose of monitoring air pollution from natural sources, mining, or industrial processes, different studies have successfully applied LIBS to characterize ensembles of aerosols [185,186,187,188,189,190,191].

**Figure 10:**
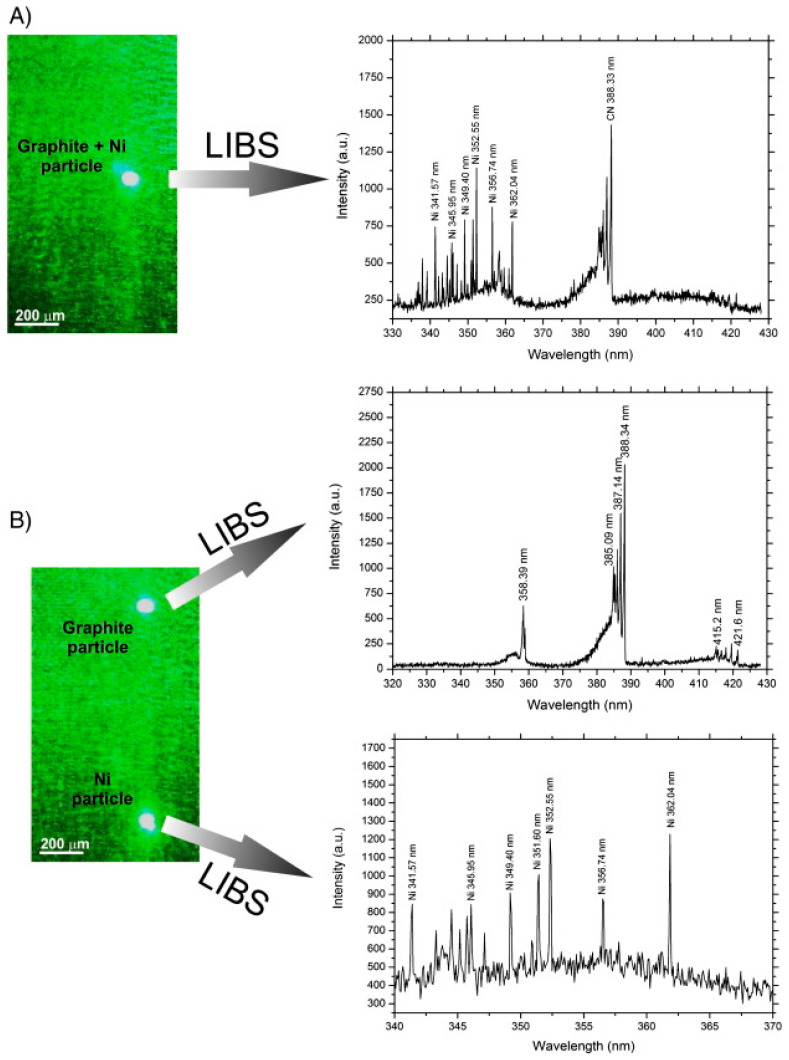
LIBS spectra of a non-homogeneous mixture from 50% graphite and 50% nickel spheres when (**A**) trapped particles are closely spaced in the same focal region and (**B**) trapped particles are properly manipulated and isolated from each other. The main emission lines are labeled in the spectra. (Reprinted with permission from [194]. Copyright (2014) Elsevier).

Prior to the incorporation of optical trapping into LIBS, Fortes et al. [192,193] demonstrated an optical catapulting-LIBS (OC-LIBS) system, where particles resting on a substrate are literally ejected into the air by a laser-induced shockwave from underneath, to get intercepted by a crossing pulsed probe beam. In comparison to conventional LIBS, this method has an advantage of higher sampling rate; however, its efficiency relies on the success of particle-LIBS beam interception, which in return depends on other characteristics such as the focusing of the catapulting beam, the material and thickness of the substrate, and the particle’s mass density [193]. An improved version combining OC-LIBS with optical trapping (OT) was first revealed in 2014, by the same group [194]. It consists in an arrangement of three lasers ensuring the main functions of the system: aerosolization (optical catapulting, Nd:YAG 1064 nm), particle trapping (Ar^+^ laser, 514.5 nm), and LIBS analysis (pulsed Nd:YAG 1064 nm). A 300 mJ pulse energy from Nd:YAG laser ensured complete breakdown of individual particles (nickel, graphite, and aluminum oxide) into plasma and the spectral analysis provided a detection efficiency of 100%. Another notable advantage of this system is the ability to, optically, separate particles inside the trap and analyze each individually (Figure 10)—a very useful approach for complex and mixed samples, like is always the case for atmospheric aerosols. An impressive sensitivity of 17 femtograms was achieved, considering that the limit of detection had been determined to be 200 attograms.

**Figure 11:**
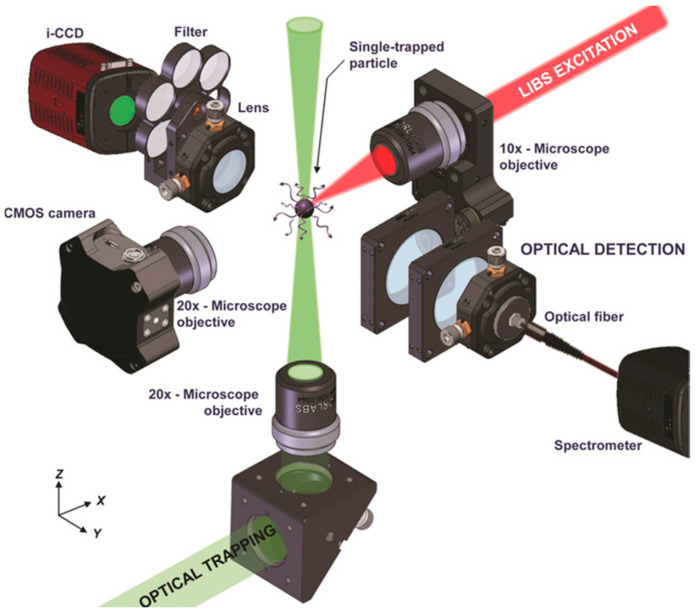
Experimental setup featuring the multiple lines of the instrument with main components labelled. Optical trapping was performed using a 532 nm Nd:YAG CW laser and a pulsed Nd:YAG at 1064 nm was used for LIBS analysis. (Reprinted with permission from [195], Copyright (2017) John Wiley and Sons).

The latest generations of OT-LIBS systems (e.g., Figure 11), with an increased excitation efficiency for small particles, where air–mass plasma acts as the main source of excitation over direct laser–particle interaction, have pushed the limit of detection down to 58.9 attograms [195] and later to an extreme sensitivity of 37 attograms [196] for copper nanoparticles. In fact, in this system, the dissociation and excitation processes are directed by heat transmission from plasma to particle and mass transfer the other way around.

Another improvement worth of mentioning is the reutilization of nanosecond pulse laser energy in single-particle LIBS by Zhou and co-workers [197]. Given that the light travels at 0.3 m ns^−1^, a 1 ns pulse can be distributed over 0.3 m in space. Upon observing that in conventional LIBS setup, a portion of the pulsed laser beam goes through the plasma unscathed, was born the idea of recycling that wasted energy by reflecting the beam in an appropriate way to come back around and overlap with the particle–plasma spot. In doing so, the emission intensity of air plasma improved by 220% under the same pulse energy. More importantly, this method works better at lower energy. For example, when the pulsed laser energy was set below the air breakdown threshold (at 10 mJ), the emission intensity of a 10 µm NaCl particle improved by 200%, with significantly reduced plasma background.

Precise positioning in OT-LIBS allows every single studied particle to be equally illuminated and vaporized. This prevents the fluctuation observed from the moving-through particle in general LIBS. The rich information from the very dense element emission lines in the spectra poses a big challenge in chemical composition identification, particularly for real-time detection and characterization of aerosol particles. In practice, the laser power stability is highly demanded, since an incomplete vaporization and poor atomization of the particle in the plasma would induce uncertainty in LIBS analysis and affect reproducibility of the experimental data.

### 3.4. Digital Holography

A single aerosol particle can be viewed by using an interferometric technique called digital holography (DH) [198], where a 3D image is reconstructed from interferences of a reference laser beam and a beam diffracted by the object. The digital in-line holography is a configuration where the object (aerosol), the reference beam, and the detector are aligned. It allows the use of the entire hologram pixel count, resulting in a high-resolution image. In practice, for small particles, the difference between measurement with and without the particle gives an intensity pattern, known as a contrast hologram, which can be expressed as follows [199,200],

$$\begin{array}{cc} I_{contrast}= & \frac{I_{holo}-I_{ref}}{I_{ref}}\\ & ≃\frac{{\left[A_{ref}\right]}^{*}A_{sca}+{\left[A_{sca}\right]}^{*}A_{ref}}{{\left|A_{ref}\right|}^{2}} \end{array}$$

where *I_ref_* and *I_holo_* are the intensities across the detector with and without the particle, *A_ref_* is the amplitude of the unscattered wave (reference wave), and *A_sca_* is the amplitude of the wave scattered by the particle (object wave).

In comparison with other 3D imaging techniques (Fourier ptychography [201,202], optical diffraction tomography [201,203], confocal imaging) requiring several measurements, DH provides the advantage of high temporal resolution, which can be useful for fast-moving particles or promptly monitoring details of physical processes.

In-line digital holography has been used to track 3D positioning of nanoparticles optically trapped in liquid [204,205,206,207]. The ability to accurately measure Brownian motions (~3.4 nm resolution) of spherical particles, in response to a change in trapping power, resulted in quantification of the trapping power threshold [204]. Hiyasaki et al. utilized DH to track the position of a 100-nm gold particle held by optical tweezers in water [205] and the recorded lateral and axial movements at low trapping power (P = 5.8 MW/cm^2^), and showed the particles had position movements with 44 nm and 69 nm in standard deviation, while they were significantly reduced to 3.0 nm and 4.5 nm at high trapping power (P = 35 MW/cm^2^).

**Figure 12:**
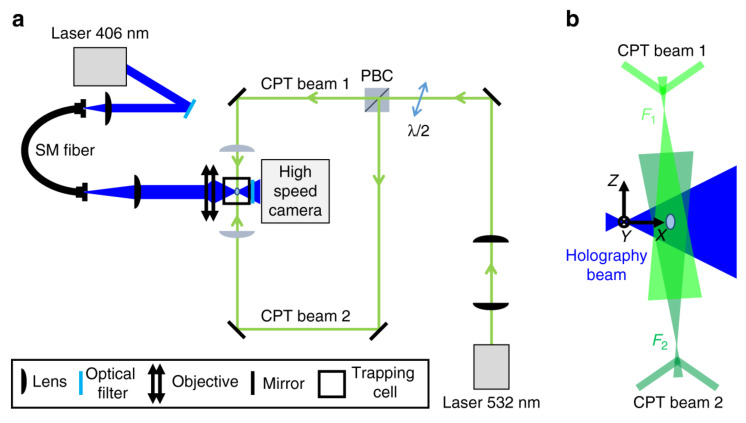
Optical trapping digital holography experimental setup. (**a**) Green lines: counter-propagating optical tweezer (CPT) for particle trapping. Blue lines: laser beam for holographic imaging. (**b**) Detail of the relative arrangement of the holography and CPT beams. The origin of the coordinate system corresponds to the focus of the holography laser. The X-axis corresponds to the propagation direction of the holography laser. The optical axis of the CPT is slightly tilted with respect to the Z-axis (reprinted from [200], with permission under Creative Commons Attribution license, Copyright (2018) Nature).

An appropriate application of DH to single-particle aerosol study was demonstrated by the dynamics of polystyrene particles in air using holographic microscopy, as shown in Figure 12 by David et al. [200]. Radii of 510 ± 60 and 800 ± 80 nm particles were experimentally retrieved in comparison to the supplier-reported values of 451 ± 6 and 800 ± 35 nm.

In addition to retrieving image, size, and position of the particle, they were also able to capture its full six-dimensional motion (three translational and three rotational)—valuable information for non-spherical particles.

**Figure 13:**
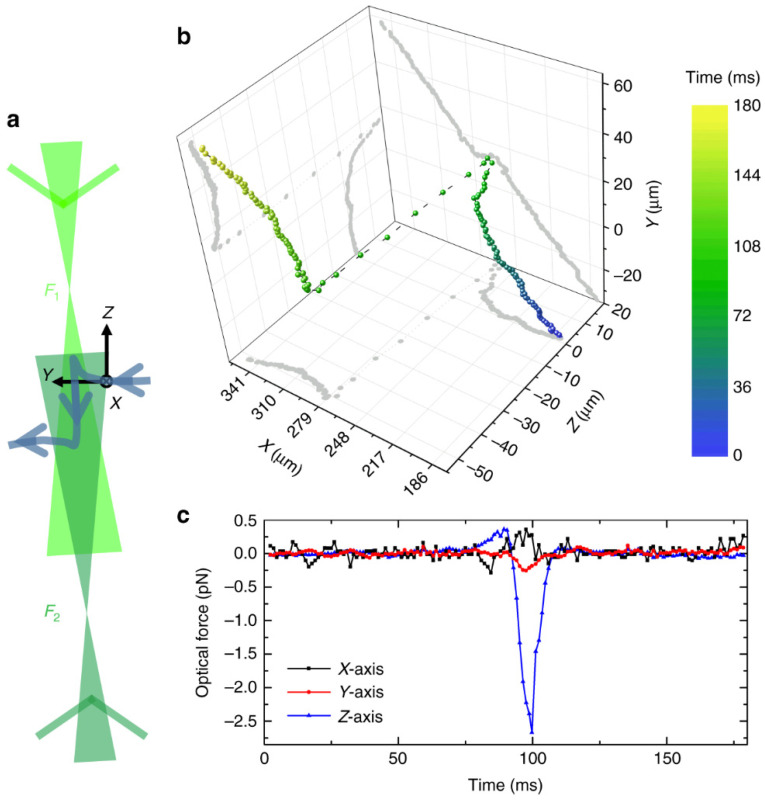
Particle motion under the influence of an external optical force. (**a**) Sketch of the trajectory (blue trace with arrows) of an 800 nm PSL sphere crossing a CPT (green laser beams). F_1_ and F_2_ are the foci of the two laser beams. The arrow on each laser beam indicates their propagation direction. The CPT is slightly tilted with respect to the Z-axis. The origin of the coordinate system corresponds to the focus of the holography laser beam. (**b**) Colored dots: Center-of-mass coordinates of the sphere as a function of time. The color code indicates the time. Gray curves: 2D projections on the respective planes. For the sake of clarity, the temporal distance between adjacent coordinate points has been chosen to be 1.2 ms. Note that the time resolution of the holograms is much better (240 μs). (**c**) Optical forces in X-direction, Y-direction, and Z-direction as a function of time (reprinted from [200], with permission under Creative Commons Attribution license, Copyright (2018) Nature).

To investigate the particle’s behavior under external forces, a steady stream of nitrogen pushed the particle across the trapping beams and a meticulous analysis of holographic images revealed detailed dynamic trajectory of the particle’s movement (direction, velocity, and acceleration), as shown in Figure 13. Holographic imaging of an irregularly shaped particle (bisphere) showed rotation motions of the particle where observed torque and rotational velocity were in a good agreement with simulations by finite-difference time-domain (FDTD).

As most imaging techniques, DH finds limitations in the quality of reconstructed image due to insufficient resolving power of the digital image sensors utilized. This issue could be a weakness for using DH to reconstruct the fine structures and surface roughness of the studied particle, which may be key features in distinguishing relatively close classes of particles. The low quality of the trapping cell could also induce unwanted side effects in measured patterns.

## 4. Summary

This article reviewed current laser technical capabilities available for single-particle aerosol characterization and its application in chemical aerosol study. Despite the complexity of the aerosol topic, laudable efforts by various researchers have significantly improved our knowledge of aerosols and opened new doors leading to better understanding of these particles. Single-particle trapping combined with a variety of laser-based analytical techniques have been used to retrieve critical chemical and physical properties (image, size, composition, morphology, refractive index, phase, reactivity, etc.) of aerosol particles for fast and accurate characterization. Recent developments in laser technology and signal processing allow resolving practical limitations, by building systems with ultra-high sensitivity and high-speed data acquisition. On the other hand, simultaneous measurements obtained by combining different techniques help get around fundamental constraints specific to each technique, with respect to the particle’s nature. Monitoring a single aerosol, in a fully controlled atmosphere, permits not only to render its physical–chemical properties, but more importantly to recognize its behavior and response toward different environmental factors, especially with respect to size-dependent processes. Only a detailed comprehension of a single-particle comportment will help minimize errors associated with averaging effects arising in ensemble measurements. Readers are encouraged to seek detailed information from studies cited in the article and to be aware of advantages resulting from combining multiple analytical techniques for an unambiguous validation of experimental observations.
